# How does social support influence autonomous physical learning in adolescents? Evidence from a chain mediation and latent profile analysis

**DOI:** 10.1371/journal.pone.0327020

**Published:** 2025-07-01

**Authors:** Jiamin Zhu, Ziyou Huang, Xiaoping Meng, Zhiyong Zhang, Xiaotong Yuan

**Affiliations:** 1 Graduate School, Shandong Sport University, Jinan, China; 2 School of Physical Education, Anqing Normal University, Anqing, China; 3 Institute of Physical Education and Social Sciences, Shandong Sports University, Jinan, China; University of Tartu, ESTONIA

## Abstract

**Purpose:**

This study examines how social support influences adolescents’ autonomous physical learning behavior, exploring the mediating roles of self-efficacy and exercise motivation, and the moderating effects of gender and behavioral typologies. The goal is to provide insights into how social support can enhance adolescents’ engagement in physical activities and inform intervention strategies.

**Methods:**

A total of 2,359 junior high school students (1,208 males and 1,151 females; mean age = 13.21 ± 0.96 years) from three public schools in Shandong Province were surveyed between October and December 2024. Participants completed the Chinese versions of the Perceived Social Support Scale (PSSS), General Self-Efficacy Scale (GSES), Motivation for Physical Activity Measure–Revised (MPAM-R), and Autonomous Physical Learning Behavior Scale (APLBS). Data were analyzed using SPSS 25.0 for descriptive statistics, Pearson correlations, t-tests, one-way ANOVAs, and hierarchical regression; Mplus 8.3 for Latent Profile Analysis (LPA); and AMOS 27.0 for Structural Equation Modeling (SEM) with bootstrapping (10,000 resamples).

**Results:**

indicated significant positive correlations among social support, self-efficacy, exercise motivation, and autonomous physical learning (all p < .01). Hierarchical regression showed that social support accounted for 27.3% of the variance in autonomous learning behavior (β = 0.524, p < .001), self-efficacy explained an additional 5.8% (β = 0.279, p < .001), and exercise motivation explained a further 4.0% (β = 0.219, p < .001), resulting in a total R^2^ = 0.373. LPA identified four behavioral profiles—Highly Engaged (26.3%), Positively Regulated (35.7%), Selectively Participative (22.2%), and Passively Participative (15.8%)—with the four-class model demonstrating optimal fit (entropy = 0.921; AIC = 37,951.9; BIC = 38,081.6; BLRT p < .001; LMR p = .0235). SEM results (CMIN/DF = 3.546; GFI = 0.970; CFI = 0.985; TLI = 0.976; NFI = 0.979; RMSEA = 0.060) showed that social support had a direct effect on autonomous learning behavior (β = 0.325, p < .001) and a total indirect effect of 0.191 (total effect = 0.516, p < .001). Specifically, self-efficacy mediated 22.19% (β = 0.114, 95% CI [0.093, 0.137]), exercise motivation mediated 11.18% (β = 0.057, 95% CI [0.043, 0.074]), and the chain pathway (social support → self-efficacy → exercise motivation → learning) accounted for 3.49% (β = 0.018, 95% CI [0.012, 0.024]) of the total effect. Multi-group SEM indicated that the SS → SE → EM mediation path was stronger for females (β = 0.127, 95% CI [0.104, 0.152]) than for males (β = 0.088, 95% CI [0.070, 0.109]). Across latent profiles, the full sequential mediation (SS → SE → EM → APLB) was significant for Positively Regulated (β = 0.074, 95% CI [0.051, 0.101]) and Selectively Participative (β = 0.066, 95% CI [0.042, 0.089]) groups, marginally significant for Passively Participative (β = 0.038, 95% CI [0.015, 0.062]), and non-significant for Highly Engaged (β = 0.013, 95% CI [–0.004, 0.031]).

**Conclusion:**

These findings demonstrate that social support enhances adolescents’ autonomous physical learning both directly and indirectly through self-efficacy and exercise motivation. Psychological resilience processes accounted for approximately 37.1% of behavioral variance, and gender differences and latent profiles moderated these pathways. Interventions should therefore focus on strengthening perceived support to boost self-efficacy and motivation—especially among female and moderately engaged students—while tailoring strategies to each behavioral profile to foster sustained autonomous learning and lifelong physical activity engagement.Keywords: Machine Learning, Neural Networks, artificial intelligence.

## 1. Introduction

With the continued implementation of the “Double Reduction” policy and the advancement of the “Healthy China” initiative, enhancing adolescents’ physical fitness and sports literacy now constitutes a cornerstone of basic education reform. Physical activity is crucial not only for adolescents’ physical health but also for their psychological well-being, social adaptation, and cultivating lifelong engagement in physical activity [1–3]. However, despite broad recognition of its importance, many adolescents demonstrate limited initiative and persistence in their physical learning. This deficiency is most pronounced in their autonomous physical learning behaviors, which remain a significant issue within current education systems [4,5].

Existing research has extensively examined the roles of social support, self-efficacy, and exercise motivation in promoting adolescent physical activity. Research has shown that social support from family, peers, and teachers positively influences participation by enhancing self-efficacy and fostering intrinsic motivation [6]. However, most studies focus on average associations, often neglect individual variability and behavioral heterogeneity among adolescents, thereby hindering our understanding of how different individuals respond to the same social support [7–10].

While earlier studies have confirmed connections between social support, self-efficacy, and motivation in influencing physical learning behaviors, there are still few thorough studies that explore these relationships in depth. Autonomous physical learning represents a multidimensional, context-sensitive construct, yet this complexity remains underexamined in current research [11,12]. Traditional analytic approaches have typically focused on linear associations, thereby overlooking the complicated interactions that characterize adolescent learning behaviors.

This study looks at this gap by using a combined SCT–SDT chain mediation framework to see how social support affects teenagers’ independent physical learning through self-efficacy and exercise motivation. It looks at how self-efficacy and exercise motivation act as middle steps and checks how they differ among various groups of behaviors. Understanding that independent physical learning is influenced by both personal motivation and outside factors, we use Latent Profile Analysis (LPA) to find different hidden groups within the sample.By using key factors—exercise motivation, self-efficacy, and perceived social support—LPA finds different groups of people with similar and different behaviors, creating a strong foundation for later analysis across multiple groups [13,14]. Finally, the study uses structural equation modeling (SEM) to look at the different psychological paths in these groups and help create specific intervention strategies.

The main new idea of this study comes from combining Social Cognitive Theory (SCT) and Self-Determination Theory (SDT) into a clear framework that looks at how social support influences teenagers’ independent physical learning through their inner motivation and confidence. It further employs Latent Profile Analysis (LPA) to delineate latent behavioral typologies and compares their underlying psychological pathways. Finally, the study uses multi-group Structural Equation Modeling (SEM) to create specific intervention strategies for each group, leading to new ideas that can improve physical education.

## 2. Research hypotheses

### 2.1. The predictive role of social support in autonomous physical learning

Autonomous physical learning behavior refers to adolescents’ tendency to independently determine the frequency, intensity, and goals of their physical activity with minimal external coercion. This self-directed engagement reflects a high level of internalized motivation and goal orientation, often considered an indicator of effective physical education and personal development [15,16].

According to Bronfenbrenner’ s ecological systems theory [17], adolescent development occurs within nested environmental systems, where micro-systems such as family, school, and peer groups exert direct influence. Social support—comprising emotional, informational, and behavioral assistance from parents, teachers, and peers—functions as a vital regulatory resource across these systems [18]. It is particularly influential in the context of physical learning, shaping adolescents’ behavioral dispositions through interpersonal reinforcement and modeling.

Empirical studies have consistently shown that perceived social support enhances adolescents’ physical activity participation by increasing initiative and persistence [19,20]. Specifically, parental encouragement and presence during physical activities significantly increase willingness to engage; teacher praise and autonomy-supportive classrooms foster greater self-efficacy; and peer group support and collective identity boost emotional attachment and behavioral continuity [21,22].

This evidence is consistent with the propositions of Social Cognitive Theory (SCT) and Self-Determination Theory (SDT). SCT emphasizes the role of self-efficacy—shaped by external modeling and encouragement—as a key trigger for behavioral action [23]. SDT explains that social support helps teenagers meet their basic psychological needs for independence, skill, and connection, which boosts their motivation and keeps them involved [24].Within this integrative framework, social support functions as a dual mechanism: it not only cultivates the belief of “I can” through enhanced competence but also nurtures the drive of “I want” by fulfilling core psychological needs.

### 2.2. The mediating role of self-efficacy

Self-efficacy refers to an individual’s cognitive appraisal of their ability to accomplish specific behavioral goals [25]. In the context of adolescent physical activity, it manifests as a student’s positive belief in their capacity to initiate, sustain, and succeed in physical engagement. Prior research has identified self-efficacy as a core intrinsic driver of adolescents’ sports participation. Specifically, it directly enhances their willingness to engage [26], increases the depth of behavioral involvement, strengthens psychological resilience when facing challenges, and elevates positive outcome expectations regarding physical activity [27].

Scholars widely recognize social support as a key antecedent in the development of self-efficacy among adolescents. Parental support, by enhancing the perceived feasibility of action, fosters positive expectations and efficacy beliefs [28]; teacher support, on the other hand, reinforces students’ sense of competence and confidence in performing physical tasks. Empirical studies have indicated that self-efficacy mediates the relationship between family support and sustained exercise behavior, indicating that external support may be internalized into self-perceptions of capability, which in turn promote long-term engagement [29].

According to Social Cognitive Theory (SCT), self-efficacy is shaped by mastery experiences, observational learning, verbal persuasion, and emotional arousal, all of which are embedded within environmental interactions [30]. Social support plays a central role in these processes by providing concrete feedback, role models, and encouragement; thereby, it strengthens cognitive beliefs about personal capability. In addition, Self-Determination Theory (SDT) suggests that when teenagers’ basic needs for independence, skill, and connection with others are met, they are more likely to accept and use outside help like social support. This positions self-efficacy as a crucial psychological mechanism linking contextual support to internalized, self-directed motivation [31].

Taken together, these theoretical and empirical insights suggest that self-efficacy may operate as a central mediating variable in the relationship between social support and autonomous physical learning behavior. Social support contributes to the construction of positive self-beliefs, while self-efficacy, in turn, activates and maintains sustained, intrinsically motivated physical engagement.

### 2.3. The mediating role of exercise motivation

Exercise motivation is a fundamental psychological construct that drives individuals to initiate and sustain engagement in physical activity. It predicts the intensity, frequency, and persistence of participation in various exercise behaviors [32]. Motivation is typically categorized into two types: intrinsic motivation, which stems from personal interest, enjoyment, and self-fulfillment; and extrinsic motivation, which arises from external rewards, social recognition, or approval [33]. The type of motivation affects how deeply and consistently people engage in activities—when someone is motivated by their own interests, they are more likely to stick with it, while motivation from outside rewards usually leads to only temporary involvement [34].

Social support has been shown to significantly enhance both the quality and magnitude of exercise motivation. According to Self-Determination Theory (SDT), when social environments meet people’s basic psychological needs for independence, skill, and connection with others, outside influences are more likely to be accepted and turned into personal motivation [35]. For adolescents, positive expectations, verbal encouragement, and emotional support from parents and teachers help clarify physical activity goals and reinforce a sense of personal efficacy and worth. These factors facilitate the internalization of motivation and contribute to the maintenance of long-term behavioral engagement in physical activity [36].

Ecological systems theory adds to this idea by highlighting that social support comes from different areas like family, school, and friends, all of which work together to influence an adolescent’s motivation [37]. Within such multilayered support systems, adolescents experience continuous modeling, reinforcement, and emotional validation, all of which contribute to the transformation of extrinsic motivators into self-endorsed forms of regulation. As this internalization process deepens, adolescents develop stronger outcome expectancies and a more autonomous orientation toward physical activity, leading to sustained and self-directed engagement.

Taken together, these theoretical and empirical insights suggest that exercise motivation may serve as a psychological conduit through which social support translates into autonomous physical learning behavior. Social support initiates motivational arousal and plays a vital role in shaping the type and internalization level of motivation, thereby influencing both the quality and duration of behavioral engagement.

### 2.4. The chain mediation role of self-efficacy and motivation

In the context of physical learning, social support influences adolescents’ autonomous physical learning behavior primarily through enhancing self-efficacy and fostering intrinsic motivation. Establishing the sequential directionality between these two mechanisms is crucial to strengthening the explanatory power of the model [38]. According to Social Cognitive Theory (SCT), self-efficacy—the belief in one’s capacity to initiate and sustain specific behaviors—serves as the proximal cognitive antecedent of action [39]. Adolescents with higher self-efficacy engage in physical activity with greater confidence and persistence [40]. More importantly, self-efficacy acts as a key mental resource that comes before motivation, helping to spark a stronger personal interest and commitment to goals [41]. From the lens of Self-Determination Theory (SDT), intrinsic motivation arises when individuals’ needs for autonomy, competence, and relatedness are satisfied. Among these, competence—closely aligned with self-efficacy—plays a pivotal role in shaping motivational quality. Only when adolescents perceive themselves as competent and capable can external encouragement and support be internalized, leading to the emergence of genuine interest and autonomous drive [42]. Thus, self-efficacy contributes directly to behavioral persistence and indirectly enhances intrinsic motivation by reinforcing the individual’s sense of agency and competence within the learning context.

Empirical research supports this sequential pathway. Studies have shown that enhanced self-efficacy leads to greater perceived task value and intrinsic goal orientation, which in turn promotes long-term engagement and persistence in physical activity [43]. For example, students who believe in their exercise capabilities are more likely to interpret effort as rewarding, internalize value-based goals, and sustain their participation through intrinsic rather than controlled motivation [44]. This sequential pattern has been demonstrated in both cross-sectional and longitudinal studies, highlighting the directional and predictive nature of the self-efficacy→motivation→behavior pathway.

From a cognitive–affective sequence perspective, self-efficacy provides the necessary belief structure upon which affective interest and motivation are built [45]. Intrinsic motivation, which is the feeling of wanting to do something because it’s enjoyable, is unlikely to happen if a person doesn’t first believe in their own abilities. As such, without a firm sense of “I can,” the emergence of “I want” remains fragile and unsustainable. Conversely, when adolescents first experience self-efficacy, they are more likely to generate sustained, self-determined motivation, resulting in more autonomous and persistent behavioral engagement [46].

### 2.5. Moderating role of gender in the indirect relationship between social support and autonomous physical learning

During adolescence, gender plays a critical role in shaping how individuals interpret and respond to social environments [47]. According to Social Cognitive Theory (SCT), the development of self-efficacy is influenced by socially mediated experiences such as verbal encouragement and observational learning. Given that female adolescents often exhibit greater relational sensitivity and receptivity to social feedback, they may be more likely to internalize supportive interactions into stronger efficacy beliefs [48]. Similarly, Self-Determination Theory (SDT) highlights that the quality of motivation relies on meeting basic psychological needs—like feeling capable, having independence, and feeling connected to others—which can be more or less important for boys and girls [49]. For instance, female students may place more emphasis on social connectedness and emotional affirmation, while male students may prioritize goal attainment and task-based validation. These gendered patterns in cognitive and emotional processing suggest that the strength of the sequential pathway from social support to motivation and behavior may differ by gender.

### 2.6. Latent typologies and structural differences in behavioral pathways

Adolescents’ autonomous physical learning behaviors display substantial heterogeneity that cannot be fully explained along a single continuous dimension. Instead, these behaviors are more accurately conceptualized as distinct latent subtypes or behavioral profiles [50]. For example, while some students engage proactively and autonomously—driven by strong intrinsic motivation and effective self-regulation—others exhibit more passive, selective, or even avoidant patterns of participation. Such variation likely reflects the complicated relationship between contextual influences and individual psychological factors [51]. Ecological systems theory posits that individual development is shaped by nested environmental systems, particularly microsystem-level influences such as family, school, and peers. Within these systems, adolescents form behavioral patterns in response to contextually situated and developmentally contingent experiences. Such development gives rise to latent behavioral heterogeneity—stable subgroups characterized by distinct constellations of psychological resources and engagement tendencies [52]. Finding these hidden subgroups helps researchers see the differences within each group, leading to a better understanding of how behaviors work.

Latent Profile Analysis (LPA) is a person-centered statistical approach used to uncover hidden sub-populations based on observed indicators. It has been widely adopted in domains such as physical activity, academic engagement, and psychological adjustment. Prior studies have demonstrated that different latent profiles exhibit systematic differences in motivational orientation, self-regulatory abilities, and perceptions of social support [53]. For instance, students in high-motivation profiles typically show sustained participation and internalized goals, whereas those in externally regulated or amotivated profiles may engage superficially or inconsistently.

Based on theories about how people interact with their environment and the idea of matching motivation, it seems likely that the ways social support, self-efficacy, and intrinsic motivation connect to different behavior patterns vary among groups. In groups where people are very motivated on their own, social support might help strengthen their confidence, making them even more motivated in a step-by-step way. In contrast, for students who are not very engaged or rely on outside influences, social support may help them get involved without really tapping into their deeper motivation. These possible differences indicate that the roles of self-efficacy and motivation can change depending on each student’s behavior and readiness to be motivated [54].

### 2.7. Present study

This study draws on Social Cognitive Theory (SCT) and Self-Determination Theory (SDT) to conceptualize how social support shapes adolescents’ autonomous physical learning behavior. SCT emphasizes the role of self-efficacy—developed through reinforcement, modeling, and encouragement—as a proximal determinant of behavioral initiation. In parallel, SDT posits that the fulfillment of basic psychological needs, particularly competence, facilitates the development of intrinsic motivation and sustained behavioral engagement.

**Fig 1:**
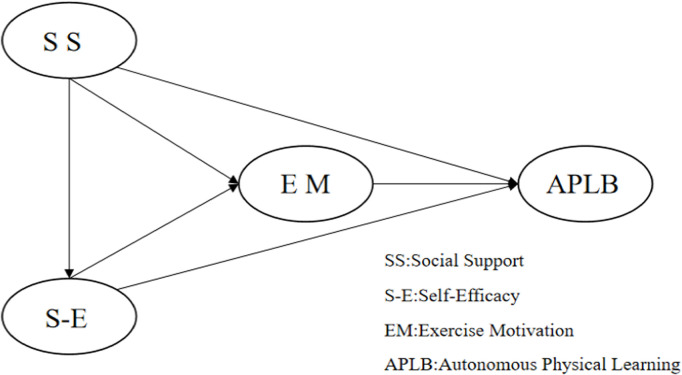
Hypothesized structural model.

Combining these ideas, we suggest that social support doesn’t directly impact behavior but works through a step-by-step mental process: it boosts self-efficacy, which then increases intrinsic motivation, and this leads to self-directed physical learning behavior. Using this framework, we create a model that shows how self-efficacy and intrinsic motivation work one after the other to connect social support with independent physical learning behavior (see Fig 1).

Therefore, this study aims to examine both the indirect mechanisms through which social support influences physical learning behavior and whether these mechanisms vary across behavioral profiles. We propose the following hypotheses:

H1: Social support has a significant positive predictive effect on autonomous physical learning behavior.
H2: Self-efficacy mediates the relationship between social support and autonomous physical learning behavior.
H3: Exercise motivation partially mediates the relationship between social support and autonomous physical learning behavior.
H4: Social support enhances self-efficacy, which in turn increases exercise motivation, thereby indirectly promoting autonomous physical learning behavior.
H5: Gender moderates the indirect effect of social support on autonomous physical learning behavior through self-efficacy and exercise motivation.
H6: The way social support affects autonomous physical learning behavior through self-efficacy and exercise motivation differs among different groups of behaviors.

## 3. Research methods

### 3.1. Participants

A stratified random sampling method was employed to recruit participants from three public junior high schools in Shandong Province, China, between October 12, 2024, and December 30, 2024. Based on an a priori power analysis conducted using G*Power 3.1, the minimum required sample size to detect a medium effect size (f² = 0.15) with 95% statistical power at a significance level of α = 0.05 was estimated to be 172 participants. The actual sample size substantially exceeded this requirement, ensuring sufficient statistical power for all planned analyses. A total of 2,475 online questionnaires were distributed, and 2,359 valid responses were received, resulting in an effective response rate of 95.31%. The sample included students from Grades 7–9, with a mean age of 13.21 years (SD = 0.96). Among them, 1,208 were male (51.2%) and 1,151 were female (48.8%).

The study was conducted in accordance with the Declaration of Helsinki. Prior to data collection, ethical approval and administrative consent were obtained from the Ethics Committee of the Institute of Sports Social Science at Shandong Sports University (Approval No. 2024019). Written informed consent was obtained from all participants after being informed about the study’s objectives, procedures, potential risks, and confidentiality measures. Participants were assured that their participation was voluntary, they could withdraw at any time without penalty, and all collected data would be kept strictly confidential and used solely for research purposes.

In addition, schoolteachers helped introduce the study to students’ parents and encouraged their involvement. After obtaining parental consent, informed consent forms were signed by the parents, and the questionnaire was completed through an online survey platform.

### 3.2. Measurement instruments

#### 3.2.1. Perceived social support.

We assessed social support using the Chinese version of the Perceived Social Support Scale (PSSS), originally developed by Zimet etl. [55] and adapted by Jiang [56]. Social support was assessed using the Chinese version of the Perceived Social Support Scale (PSSS) originally developed by Zimet et al. and adapted by Jiang. The scale consists of 12 items across two sub-dimensions: “family support” and “support outside the family,” rated on a 7-point Likert scale (1 = strongly disagree, 7 = strongly agree). Higher scores indicate greater perceived support. Confirmatory factor analysis (CFA) showed a satisfactory model fit in the current sample (CFI = 0.95, TLI = 0.94, RMSEA = 0.046), with excellent internal consistency (Cronbach’s α = 0.966) and factor loadings ranging from 0.76 to 0.78.

#### 3.2.2. General self-efficacy.

Self-efficacy was measured using the Chinese version of the General Self-Efficacy Scale (GSES), developed by Schwarzer and Jerusalem [57] and adapted by Wang et al. [58]. This 10-item scale uses a 4-point Likert response format (1 = not at all true, 4 = exactly true), with higher scores indicating greater self-efficacy. In this study, the scale was very reliable, with a Cronbach’s α of 0.937, factor loadings between 0.65 and 0.85, an average variance extracted of 0.60, and a composite reliability of 0.94. Model fit was acceptable (CFI = 0.94, RMSEA = 0.051).

#### 3.2.3. Motivation for physical activity.

Exercise motivation was assessed using a short version of the Motivation for Physical Activity Measure – Revised (MPAM-R), developed by Ryan et al. [59] and adapted by Chen et al. [60]. The scale contains 15 items measured on a 5-point Likert scale (1 = strongly disagree, 5 = strongly agree), assessing the strength of both intrinsic and extrinsic motivations for participating in physical activity. The scale demonstrated strong psychometric properties in this study (Cronbach’s α = 0.954; factor loadings: 0.73–0.84; CR = 0.89; CFI = 0.96; RMSEA = 0.045).

#### 3.2.4. Autonomous physical learning behavior.

Autonomous physical learning behavior was measured using the 19-item scale developed by Wu [61]. Responses were rated on a 5-point Likert scale (1 = not at all true, 5 = entirely true). The scale demonstrated high internal consistency (Cronbach’s α = 0.933) and structural validity, with factor loadings between 0.65 and 0.74, CR = 0.87, CFI = 0.94, and RMSEA = 0.043.

### 3.3. Data analysis procedures

Data were analyzed using SPSS 25.0, Mplus 8.3, and AMOS 27.0. First, Cronbach’s α coefficients were used to check how reliable each scale was, and confirmatory factor analysis (CFA) was done to see if the measurement tools were valid. Model fit was evaluated based on commonly accepted criteria: comparative fit index (CFI) > 0.90, Tucker–Lewis index (TLI) > 0.90, and root mean square error of approximation (RMSEA) < 0.08.

Descriptive statistics and Pearson correlation analyses were conducted in SPSS to examine the distributions of key variables and their interrelationships. In addition, independent samples t-tests and one-way analysis of variance (ANOVA) were used to explore differences in key psychological variables across demographic groups. A multiple linear regression analysis was also done to see how social support, self-efficacy, and exercise motivation affect adolescents’ ability to learn physical activities on their own, and to understand how important each of these factors is.

To better understand the differences in autonomous physical learning behavior among middle school students, a Latent Profile Analysis (LPA) was done using 19 items from the Autonomous Physical Learning Scale as continuous indicators. This method of grouping people, based on a statistical model, sorts individuals into categories that share similar behavior traits. To check how well the model fits, several measures were used, including the Akaike Information Criterion (AIC), Bayesian Information Criterion (BIC), adjusted BIC (aBIC), entropy, and likelihood tests like the Lo–Mendell–Rubin Likelihood Ratio Test (LMR-LRT) and the Bootstrap Likelihood Ratio Test (BLRT). Models were created with one to five groups, and the best number of groups was chosen based on how well they fit the data and how easy they were to understand.

We conducted multi-group structural equation modeling (SEM) in AMOS after profile identification, using the latent profiles derived from LPA as grouping variables. The analysis tested whether the mediation pathway differed across groups. Mediation effects were assessed using the bootstrap method with 10,000 resamples, and 95% confidence intervals were constructed to determine statistical significance. The significance level was set at α = 0.05.

## 4. Results

### 4.1. Test for common method bias

Harman’ s single-factor test was performed to assess the presence of common method bias in the present study by conducting an unrotated exploratory factor analysis on all measurement items.The results extracted seven factors with eigenvalues greater than 1. The first factor accounted for 36.73% of the total variance, which is below the empirical threshold of 40% [62], indicating that common method bias was not a significant concern. The result suggests that the measurement instruments used in this study possessed good discriminant validity.

### 4.2. Descriptive statistics and correlational analysis

Table 1 presents the descriptive statistics and Pearson correlations among the four focal constructs. The means (standard deviations) were as follows: autonomous physical learning behavior, M = 3.91 (SD = 0.75); perceived social support, M = 5.52 (SD = 1.27); self-efficacy, M = 2.72 (SD = 0.76); and exercise motivation, M = 3.85 (SD = 0.85). All inter-variable correlations were positive and statistically significant (p < .01). Notably, social support correlated most strongly with autonomous learning behavior (r = .52), while self-efficacy demonstrated robust associations with social support (r = .51) and with autonomous learning (r = .47). Exercise motivation also showed moderate positive correlations with autonomous learning (r = .42), self-efficacy (r = .33), and social support (r = .38). These patterns provide preliminary evidence that self-efficacy and exercise motivation may mediate the relationship between social support and students’ autonomous physical learning behavior.

**Table 1:** Descriptive statistics and correlation matrix of main variables.

Variables	M ± SD	APLB	S S	S-E	EM
**APLB**	3.91 ± 0.75	—			
**S S**	5.52 ± 1.27	0.52^**^	—		
**S-E**	2.72 ± 0.76	0.47^**^	0.51^**^	—	
**E M**	3.85 ± 0.85	0.42^**^	0.38^**^	0.33^**^	—

Note: M = Mean; SD = Standard Deviation; p < 0.01 **: All correlation coefficients are significant at the 0.01 level APLB = Autonomous Physical Learning Behaviors; S S = Social Support; S-E = Self-Efficacy; E M = Exercise Motivation

### 4.3. Group comparisons using t-tests and ANOVAs

Independent-samples t-tests and one-way ANOVAs examined whether autonomous physical learning (APL), social support (SS), self-efficacy (SE), and exercise motivation (EM) varied by gender, grade, boarding status, school location, and parental education. Male students reported significantly higher scores than female students on all four constructs—APL (M_male = 3.95, SD = 0.73 vs. M_female = 3.88, SD = 0.76), SS (5.61 vs. 5.44), SE (2.77 vs. 2.66), and EM (3.89 vs. 3.82)—with t(2357) values ranging from 2.05 to 3.55, all p < .05. A one-way ANOVA indicated a modest but significant decline in SS across grade levels, F(2, 2356) = 6.44, p < .001, as 7th-graders reported the highest support (M = 5.62, SD = 1.24) and 9th-graders the lowest (M = 5.38, SD = 1.31). Exercise motivation also decreased by grade, F(2, 2356) = 4.25, p = .015, with 7th-graders scoring M = 3.91 (SD = 0.83) and 9th-graders M = 3.78 (SD = 0.86). No significant differences by grade were found for APL or SE (both p > .10). Boarding status and parental education showed no significant associations with any of the four constructs (all p > .20). Finally, rural students reported slightly higher SE (M = 2.76, SD = 0.77 vs. 2.68, SD = 0.75) and EM (M = 3.91, SD = 0.86 vs. 3.81, SD = 0.84) than their urban peers, t(2357) = 2.49 and 2.79, respectively, both p < .02, while APL and SS did not differ by school location (both p > .10) (Table 2).

**Table 2:** Group differences in APL, SS, SE, and EM by demographic variables.

Variable	n (%)	APIB		S S		S-E		EM	
		M	t/F	M	t/F	M	t/F	M	t/F
**Gender**									
** Male**	1208 (51.2%)	3.88	**2.19** **(0.02)**	5.44	**3.23** **(0.01)**	2.66	**3.34** **(0.01)**	3.82	**2.01** **(0.04)**
** Female**	1151 (48.8%)	3.95		5.61		2.77		3.89	
**Grade**									
** 7th grade**	786 (33.3%)	3.94	2.24 (0.11)	5.62	**6.44** **(0.01)**	2.74	**4.25** **(0.02)**	3.91	**3.18** **(0.04)**
** 8th grade**	970 (41.1%)	3.92		5.53		2.74		3.84	
** 9th grade**	603 (25.6%)	3.86		5.38		2.64		3.78	
**Boarding status**									
** Non-boarder**	1573 (66.7%)	3.93	1.84 (0.06)	5.56	**2.03** **(0.04)**	2.73	0.88 (0.37)	3.86	0.56 (0.57)
** Boarder**	786 (33.3%)	3.87		5.44		2.69		3.84	
**School location**									
** Rural**	940 (39.9%)	3.93	1.08 (0.28)	5.57	1.54 (0.13)	2.76	**2.49** **(0.01)**	3.91	**2.79** **(0.01)**
** Urban**	1419 (60.1%)	3.90		5.49		2.68		3.81	
**Parental education**									
** Below bachelor’s**	1300 (55.3%)	3.92	0.64 (0.52)	5.54	0.85 (0.39)	2.73	1.24 (0.21)	3.86	0.89 (0.37)
** Bachelor’s or above**	1054 (44.7%)	3.91		5.49		2.69		3.83	

**Note.** Values represent group means. Standard deviations (SD) are reported in the main text.Bolded means indicate statistically significant group differences based on independent-samples t-tests (t(2357)) and one-way ANOVAs (F(2, 2356)).APL = Autonomous Physical Learning; SS = Social Support; SE = Self-Efficacy; EM = Exercise Motivation.

### 4.4. Regression analysis

We conducted a four-step hierarchical regression analysis to identify the key predictors of adolescents’ autonomous physical learning behavior. In Model 0, which only looked at demographic factors like gender and grade, the amount of explained variance was very small (R² = 0.002; F(2, 2356) = 4.76, p = .029), with gender being a weak positive predictor (β = 0.068, t = 2.18, p < .05) and grade having no significant impact (β = –0.029, t = –1.37, ns). Model 1 added social support, which greatly improved the ability to explain the results (ΔR² = 0.273, p < .001), bringing the total to R² of 0.275 (F(3, 2355) = 447.53, p < .001). Social support exerted a strong positive influence (β = 0.524, t = 29.81, p < .001), and the effects of gender and grade became non-significant. In Model 2, when self-efficacy was added, it explained an extra 5.8% of the differences (ΔR² = 0.058, p < .001), increasing the total R² to 0.333 (F(4, 2354) = 392.55, p < .001); both social support (β = 0.384, t = 19.70, p < .001) and self-efficacy (β = 0.279, t = 14.32, p < .001) were important factors. Finally, Model 3 included exercise motivation, which explained an additional 4.0% of variance (ΔR² = 0.040, p < .001) and resulted in a final R² of 0.373 (F(5, 2353) = 350.45, p < .001). In this full model, social support (β = 0.323, t = 16.51, p < .001), self-efficacy (β = 0.238, t = 12.38, p < .001), and exercise motivation (β = 0.219, t = 12.24, p < .001) each made unique and significant contributions, while gender and grade remained non-significant throughout. These results indicate that, aside from minor demographic factors, having more social support, believing in oneself more, and being more motivated to exercise each independently lead to greater involvement in physical learning (Table 3).

**Table 3:** Hierarchical multiple regression predicting students’ autonomous physical learning behavior.

Predictor Variables	Model 0		Model 1		Model 2		Model 3	
	β	t	β	t	β	t	β	t
**Gender**	0.068	2.182*	0.010	0.578	0	0.019	−0.002	−0.112
**Grade**	–0.029	–1.372	−0.01	−0.489	−0.006	−0.292	0.002	0.111
**Social Support**			0.524	29.808**	0.384	19.700**	0.323	16.512**
**Self-Efficacy**					0.279	14.320**	0.238	12.376**
**Exercise Motivation**							0.219	12.238**
**Model Significance**	p = 0.029		p < 0.001		p < 0.001		p < 0.001	
**R²**	0.002		0.275		0.333		0.373	
**△R²**	–		0.273		0.058		0.040	
**F**	4.760		447.525		392.547		350.450	

Note. β = standardized regression coefficient; t = t-value; R² = coefficient of determination; ΔR² = incremental variance explained compared to the previous model.Model 0 includes gender and grade as demographic controls. Model 1 adds social support, Model 2 adds self-efficacy, and Model 3 adds exercise motivation. All ** coefficients and F-values are significant at the p < .01 level.

### 4.5. Structural equation modeling and mediation effect testing

**Fig 2:**
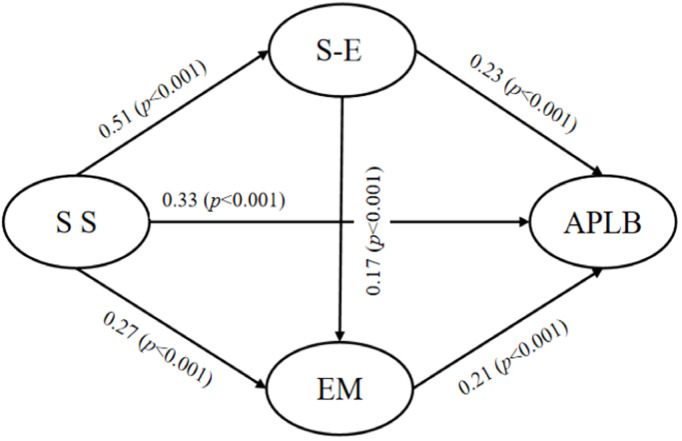
Structural equation model of social support, self-efficacy, exercise motivation, and autonomous physical learning.

To understand how social support helps middle school students become more active by boosting their self-confidence and motivation to exercise, we built a structural equation model (SEM) using AMOS 27.0, following the mediation testing steps from Wen et al. with maximum likelihood estimation. Gender and grade were included as covariates [63], and bootstrapping was employed to assess mediation effects. The model fit was excellent (CMIN/DF = 3.546, GFI = 0.97, CFI = 0.985, TLI = 0.976, NFI = 0.979, RMSEA = 0.06), supporting subsequent path analyses. The overall effect of social support on independent physical learning was 0.4326, which includes a direct effect of 0.276 and an indirect effect of 0.1566. This means that if perceived social support increases by one standard deviation, learning behavior is expected to increase by 0.4326 standard deviations, with about 36% of this effect happening through psychological factors. Specifically, self-efficacy influenced 22.19% of the effect, exercise motivation influenced 11.18%, and the connection from self-efficacy to exercise motivation influenced 3.49%. The direct pathway (β = 0.325) is substantively meaningful when these standardized effects are interpreted against behavioral research benchmarks (β ≈ 0.3 as moderate). Pairwise comparisons further revealed that the indirect effect through exercise motivation exceeded that through self-efficacy (Δ = 0.0218, 95% CI [0.0103, 0.0321]), while the chain mediation effect was significantly smaller than both the exercise motivation (Δ = –0.0658, 95% CI [–0.0831, –0.0482]) and the self-efficacy pathways (Δ = –0.0440, 95% CI [–0.0583, –0.0269]) (Fig 2) (Table 4).

**Table 4:** Direct, indirect, and total effects of social support on autonomous physical learning with bootstrap confidence intervals.

Model Pathways	Effect Size	Effective quantity	Boot SE	Bootstrap95%CI	
				Lower	Upper
**Path1**	0.114	22.19%	0.010	0.093	0.137
**Path 2**	0.057	11.18%	0.007	0.043	0.074
**Path 3**	0.018	3.49%	0.003	0.012	0.024
**Direct effect**	0.325	63.14%	0.022	0.284	0.368
**Total Indirect Effect**	0.191	–	–	–	–
**Total Effect**	0.516	100	0.018	0.481	0.551

Note: Path 1 = Social Support → Self-Efficacy → Autonomous Physical Learning;Path 2 = Social Support → Exercise Motivation → Autonomous Physical Learning;Path 3 = Social Support → Self-Efficacy → Exercise Motivation → Autonomous Learning

### 4.6. Multi-group test of the moderating effect of gender

Our analysis showed that when we included psychological factors like social support, self-efficacy, and exercise motivation, the impact of demographic factors like gender and grade became almost unimportant, with their effects being very small. This pattern underscores that these psychological factors explain markedly more variance in autonomous physical learning than do basic demographic characteristics. Based on these findings, we used multi-group structural equation modeling (SEM) to see if gender affects how social support, self-efficacy, and exercise motivation influence autonomous learning, and to compare the strength of these relationships between boys and girls. Before making these comparisons, we confirmed that the measurements for the four key areas (autonomous physical learning, social support, self-efficacy, and exercise motivation) were consistent by testing different models as outlined by Vandenberg and Lance [64]. All changes in ΔCFI and ΔRMSEA were within the accepted limits (ΔCFI < 0.01; ΔRMSEA < 0.015), confirming that multi-group SEM is appropriate for analyzing how gender affects the results. Table 5 shows the results for the autonomous physical learning scale, and you can find detailed information for the other constructs in Appendix Tables A1–A3 (S1 File).

**Table 5:** Measurement invariance of the autonomous physical learning scale across gender.

	χ2(df)	CFI	TLI	RMSEA[95% CI]	△CFI	△TLI	△RMSEA
**Configurl**	1260.529 (146)	0.937	0.930	0.079 [0.075,0.084]			
**Metric**	1271.899 (161)	0.936	0.931	0.078 [0.074,0.083]	0.001	0.001	0.001
**Scalar**	1288.123 (179)	0.935	0.930	0.076 [0.072,0.081]	0.001	0.001	0.002

To look at how gender affects the relationships between different factors, we did more multi-group SEM and shared the indirect and total effects in Table 6. Results indicated that the mediation path from social support through self-efficacy to exercise motivation (SS → SE → EM) was significantly stronger for female students (β = 0.127, 95% CI [0.104, 0.152]) compared to males (β = 0.088, 95% CI [0.070, 0.109]). The finding suggests that girls may rely more heavily on internal self-belief mechanisms to translate social support into motivational outcomes. Detailed structural path comparisons across gender (including χ² difference tests) are reported in Appendix Table A4 (S1 File).

**Table 6:** Comparison of indirect and total effects across gender.

Path	Male β [95% CI]	Female β[95% CI]	Gender Difference
**SS → SE → EM**	0.088[0.070–0.109]	0.127 [0.104–0.152]	Supported
**SE → EM → APLB**	0.041 [0.025–0.060]	0.046 [0.028–0.068]	Not supported
**SS → SE → EM → APLB**	0.020 [0.013–0.030]	0.024 [0.015–0.036]	Not supported
**SS → EM → APLB**	0.062 [0.045–0.083]	0.055 [0.037–0.075]	Not supported
**Total indirect effect**	0.171	0.206	–
**Direct effect**	0.310	0.347	–
**Total effect**	0.481	0.553	–

**Note:** SS = Social Support;S-E = Self-Efficacy;EM = Exercise Motivation;APLB = Autonomous Physical Learning Behavior

To examine gender-based differences in the mediation structure, we conducted a multi-group SEM analysis and compared the indirect and total effects (see Table 6). The SS → SE → EM pathway was much stronger for female students (β = 0.127, 95% CI [0.104, 0.152]) compared to male students (β = 0.088, 95% CI [0.070, 0.109]), which means that girls are more likely to use their self-belief to turn social support into motivation for exercise. Other indirect pathways—SE → EM → APL, SS → SE → EM → APL, and SS → EM → APL—showed no significant gender differences, as their confidence intervals overlapped. Moreover, the overall indirect effect (β_female = 0.347 vs. β_male = 0.310) and total effect (β_female = 0.553 vs. β_male = 0.481) were slightly larger for females, indicating a modest but meaningful gender difference (Δβ = +0.072). Detailed χ² difference tests for all structural paths are provided in Appendix Table A4 (S1 File).

### 4.7. Latent profile analysis of autonomous physical learning

Even though the gender-based multi-group SEM showed a notable difference in how self-efficacy affects exercise motivation, the overall connection between social support, self-efficacy, exercise motivation, and independent physical learning was mostly the same for both boys and girls. This finding implies that gender alone does not fully account for the subtle heterogeneity in how social support is translated into self-regulated physical learning. To uncover more psychologically meaningful subpopulations, we turned to a person-centered approach—Latent Profile Analysis (LPA). Unlike grouping by demographics, LPA classifies individuals according to their own patterns on the 19-item autonomous physical learning scale, thereby revealing a finer-grained variation in motivational and regulatory profiles. These groups we found based on data provide a strong basis for checking if the ways we think certain factors influence behavior work differently for different types of people. Using Mplus 8.3, we tested different LPA models with one to five groups, checking each option with several measures: the Akaike Information Criterion (AIC), Bayesian Information Criterion (BIC), sample-size adjusted BIC (aBIC), entropy (with values over 0.80 showing good accuracy), the Lo–Mendell–Rubin adjusted likelihood ratio test (LMRT), and the Bootstrap Likelihood Ratio Test (BLRT). Although the five-class solution produced marginally lower information criteria, one of its classes comprised only 3.5% of the sample and did not differ meaningfully on key variables. So, we chose the four-class model because it had a good fit with the data (entropy = 0.85; LMRT and BLRT both p < .001), made sense theoretically, and was useful in practice. The detailed LPA comparison is summarized in Table 7.

**Table 7:** Fitting index and group size of latent profile analysis models.

Indices	Unconditional Model				
	1-Profile	2-Profile	3-Profile	4-Profile^a^	5-Profile
**Fit statistics**					
** AIC**	44961.1	41120.0	39215.0	**37951.9**	37677.4
** BIC**	45006.2	41193.4	39316.5	**38081.6**	37835.3
** aBIC**	44980.8	41152.1	39259.3	**38008.5**	37746.4
** Entropy**	1	0.837	0.894	**0.921**	0.923
** BLRT**	–	0.0000	0.0000	**0.0000**	0.0000
** LMR**	–	0.0000	0.0051	**0.0235**	0.1311
**Group size (%)**					
** C1**	(2078)100.0	808 (38.8%)	864 (41.5%)	**106 (5.2%)**	98 (4.8%)
** C2**	–	1270 (61.2%)	401 (19.2%)	**742 (35.7%)**	462 (22.2%)
** C3**	–	–	813 (39.3%)	**770 (37%)**	745 (35.8%)
** C4**	–	–	–	**460 (22.1%)**	702 (33.7%)
** C5**	–	–	–	**–**	71 (3.5%)

Note: AIC = Akaike Information Criterion; BIC = Bayesian Information Criterion; aBIC = adjusted Bayesian Information Criterion; LMRT = Lo–Mendell–Rubin adjusted likelihood‐ratio test; BLRT = Bootstrap likelihood‐ratio test. a The best‐fit solution is indicated in bold.

**Fig 3:**
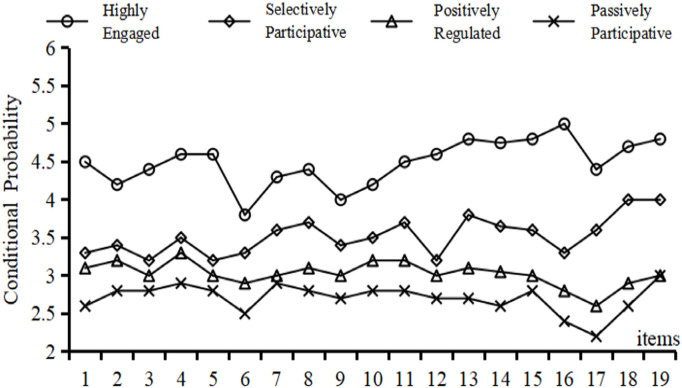
Mean response curves across four latent profiles of autonomous physical learning.

Based on the four‐class solution, we identified four distinct profiles of autonomous physical learning: (1) Highly Engaged, characterized by consistently strong intrinsic motivation and proactive self‐regulation; (2) Positively Regulated, marked by high self‐efficacy and moderate intrinsic drive; (3) Selectively Participative, in which students engage primarily in response to external cues or specific interests; and (4) Passively Participative, featuring low motivational intensity and minimal self‐directed involvement (see Fig 3). Together, these profiles reveal substantial heterogeneity in both the behavioral engagement and underlying motivational processes among middle school students.

**Fig 4:**
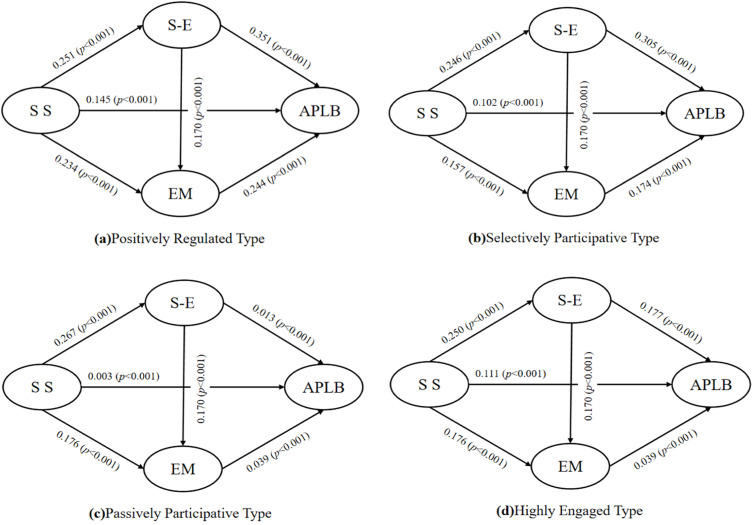
Multi-group structural equation models for four latent types of autonomous physical leaming.

To see if the ways social support, self-efficacy, exercise motivation, and autonomous physical learning are connected vary by different behavior types, we used a multi-group SEM with the four groups we identified (Highly Engaged, Positively Regulated, Selectively Participative, and Passively Participative) as categories (Fig 4). After confirming that our measurements were consistent across groups, we compared a model that allowed all relationships to change with a model that kept them the same for all groups. The constrained model fit significantly worse (Δχ²(8) = 52.42, p < .001) (Table 8).

**Table 8:** Model fit indices for measurement invariance testing across.

Model Type	CFI	RMSEA	ΔCFI	ΔRMSEA
**Configural Invariance Model**	0.975	0.045	–	–
**Metric Invariance Model**	0.974	0.046	0.001	0.001
**Scalar Invariance Model**	0.958	0.061	0.016	0.015
**Model fit criteria**	≥ 0.90	≤0.08	< 0.01	< 0.015

Bootstrapped indirect effects (10,000 resamples; Table 9) revealed that the chain mediation path (SS → SE → EM → APL) was strong and significant in the Positively Regulated (β = 0.074, 95% CI [0.051, 0.101]) and Selectively Participative (β = 0.066, 95% CI [0.042, 0.089]) profiles. In the Passively Participative group, only the two-step SS → SE → APL path remained significant (β = 0.061, 95% CI [0.035, 0.086]), reflecting a weakened SE → EM link. For the Highly Engaged group, neither the direct nor indirect chain effects reached significance (β = 0.013, 95% CI [–0.004, 0.031]), indicating minimal responsiveness to external support.

**Table 9:** Indirect effects of the chain mediation pathway by profile type.

Profile Type	Effect Size	Bootstrap95%CI	
		Lower Upper	
**Highly Engaged Type**	0.013	−0.004	0.031
**Positively Regulated Type**	0.074	0.051	0.101
**Selectively Participative Type**	0.066	0.042	0.089
**Passively Participative Type**	0.038	0.015	0.062

These results show that the strength and existence of both direct and indirect connections differ greatly among different groups, providing strong evidence that various ways of independent physical learning are supported by different psychological factors.

## 5. Discussion and interpretation

This study integrates Social Cognitive Theory (SCT) and Self-Determination Theory (SDT) within a sequential “support → beliefs → motivation → behavior” framework. According to SCT, self-efficacy constitutes the core belief for initiating and maintaining behavior, whereas SDT posits that satisfying the competence need is a prerequisite for intrinsic motivation. In this study, social support boosts self-efficacy by providing examples to follow (from the SCT viewpoint) and meets the needs for competence, autonomy, and relatedness (from the SDT viewpoint), which helps people develop their own motivation and act on it independently. Consequently, this research not only confirms the reciprocal enhancement between self-efficacy and intrinsic motivation but also elucidates how external support, while satisfying basic psychological needs, reinforces the belief system. These findings provide robust theoretical and empirical support for the proposed support → beliefs → motivation → behavior model. The study also employs Latent Profile Analysis (LPA) to identify distinct behavioral profiles in autonomous physical learning among middle school students, thereby revealing underlying psychological mechanismss and group-level heterogeneity.

### 5.1. The crucial role of social support in autonomous physical learning

This study demonstrates a significant and substantial positive association between social support and adolescents’ autonomous physical learning behavior, supporting Hypothesis 1. The standardized direct effect was β = 0.33, with a corresponding Cohen’s f² ≈ 0.38, indicating a large effect size based on established benchmarks. In practical terms, this means that a one-standard-deviation increase in perceived social support predicts an average increase of 0.33 standard deviations in students’ engagement in self-directed physical activity. This level of influence is behaviorally meaningful, as it may lead to more frequent voluntary participation, stronger goal commitment, and improved persistence in physical learning contexts. This finding agrees with earlier studies that show social support is important for how teenagers get involved, but this study goes further by providing a combined explanation related to physical education [28,65].

From the perspective of social cognitive theory, social support enhances adolescents’ beliefs in their own behavioral capabilities through mechanisms such as reinforcement, observational learning, and verbal encouragement. This enhancement of perceived competence promotes both the initiation and maintenance of physical learning behavior. Meanwhile, self-determination theory emphasizes that when adolescents’ psychological needs for autonomy, competence, and relatedness are fulfilled, they are more likely to develop intrinsic motivation and maintain sustained behavioral engagement. Our findings suggest that social support helps create a positive environment and also acts as a source of motivation that people take in through their thoughts and feelings, which strengthens their ability to manage their own physical learning [66].

In addition, the results are consistent with Bronfenbrenner’s ecological systems theory, which views adolescent development as the outcome of ongoing interactions within nested social environments. Different forms of social support—originating from parents, teachers, and peers—play distinct roles in shaping adolescents’ motivational experiences. Parental involvement offers emotional security and promotes a sense of personal value [67]. Teacher guidance and constructive feedback increase the perceived relevance and value of physical tasks [68]. Peer affiliation fosters a sense of belonging, enhancing participation through shared group identity and emotional resonance [69]. These types of support operate interactively across social settings to strengthen adolescents’ autonomous engagement in physical learning.

### 5.2. The partial mediating role of self-efficacy

This study finds that self-efficacy partially mediates the relationship between social support and adolescents’ autonomous physical learning behavior, providing empirical support for Hypothesis 2. The indirect effect through self-efficacy accounted for 22.19% of the total effect, with a standardized coefficient of β = 0.114 and a corresponding Cohen’s f² ≈ 0.08, indicating a small to medium practical effect. In behavioral terms, the evidence suggests that a one-standard-deviation increase in perceived social support leads to an average gain of 0.114 standard deviations in self-directed physical learning through self-efficacy alone. While this advantage may appear modest, it reflects a meaningful psychological shift from external encouragement to internal belief in capability, which can sustain engagement across varying physical learning contexts.

This finding builds on and expands Social Cognitive Theory (SCT), which highlights self-efficacy as a key factor in starting behaviors, managing effort, and sticking with challenges [70]. In line with Bandura’ s efficacy-expectation framework, social support contributes to the development of efficacy beliefs through mastery experiences, verbal persuasion, and vicarious learning. For example, parents’ praise of effort, teachers’ constructive feedback, and successful task completion experiences reinforce adolescents’ confidence in their ability to succeed (“I can do it”), which in turn strengthens their task persistence, goal setting, and self-monitoring behaviors. Our findings build on this theory by showing that self-efficacy is the mental link that helps people turn outside signals into lasting independent physical activity [71].

Beyond its role as a belief, self-efficacy should be understood as a dynamic self-regulatory system. Rather than being a static trait, it actively governs how adolescents transform environmental input into personal action plans and motivational direction [72]. High self-efficacy enables adolescents to translate social support into concrete self-management strategies, such as goal setting, effort calibration, and recovery from setbacks. From a practical perspective, this implies that educators and parents should offer verbal support and design developmentally appropriate challenge levels, reinforce incremental success, and provide individualized feedback that affirms perceived competence [73]. These strategies can repeatedly reaffirm adolescents’ sense of control and mastery, reinforcing their sustained and self-determined participation in physical learning.

In summary, self-efficacy functions as a cognitive–motivational converter: it internalizes social support into belief systems and action plans that energize voluntary engagement. Interventions that aim to enhance adolescents’ autonomous physical learning should focus not only on increasing available support but also on activating and maintaining adolescents’ internal efficacy cycles. This finding offers both theoretical clarity and applied insight, illustrating the importance of multi-level educational structures that repeatedly stimulate self-efficacy through authentic experience, reflection, and feedback.

### 5.3. The partial mediating role of exercise motivation

This study finds that exercise motivation partially mediates the relationship between social support and autonomous physical learning behavior, providing support for Hypothesis 3. The indirect effect through motivation accounted for 11.18% of the total effect (β = 0.057, p < .01), with a corresponding Cohen’s f² ≈ 0.02, indicating a small but meaningful effect size. Although modest in magnitude, this effect reflects a critical mechanism by which perceived social support is converted into sustained self-directed engagement. In practical terms, this suggests that increasing social support does not simply raise motivation levels but enhances the quality of motivation—fostering more internalized, value-driven engagement in physical activity [74].

This finding aligns with the core principles of Self-Determination Theory (SDT), which asserts that social environments promote intrinsic motivation when they satisfy individuals’ basic psychological needs for autonomy, competence, and relatedness. Emotional support, encouragement, and shared goal-setting from parents, teachers, and peers reinforce adolescents’ perceptions of personal value and self-endorsement of activity goals [75]. These experiences help adolescents move from “I should do this” to “I want to do this,” reflecting a qualitative shift toward self-determined motivation. Social support thus functions not merely as an external prompt but as a psychological activator that transforms contextual cues into internal motivational energy.

Moreover, our results highlight that different sources of social support facilitate motivation through distinct psychological pathways. Parental support promotes identity congruence with physical activity by reinforcing exercise-related values and routines [76]. Teacher support provides structured competence-building experiences through clear performance expectations and feedback. Peer support, particularly salient during adolescence, contributes to emotional resonance and self-comparison, strengthening social belonging and relatedness. These differentiated pathways converge to enhance the self-regulatory quality of motivation, equipping adolescents with the internal resources needed to maintain effort and interest over time [77].

In summary, this study reveals that exercise motivation operates as a motivational conduit between environmental support and behavioral autonomy [78]. By improving motivational quality rather than merely increasing quantity, social support enhances adolescents’ capacity for persistent, self-endorsed engagement. These findings demonstrate the value of cultivating activity environments that simultaneously support autonomy, competence, and relatedness. Interventions that intentionally embed these psychological affordances are more likely to foster sustainable physical learning behavior through the motivational pathway.

### 5.4. The chain mediation mechanism of self-efficacy and motivation

This study shows that self-efficacy and exercise motivation act as steps in the process that connects social support to independent physical learning behavior, backing up Hypothesis 4 with real evidence. Although the chain mediation effect accounted for only 3.49% of the total effect (β = 0.018, 95% CI [0.008, 0.031]; Cohen’s f² ≈ 0.01), its educational significance should not be underestimated. A one-standard-deviation increase in perceived social support led to a 0.018-standard-deviation increase in learning behavior through this sequential pathway. Even though it’s a small number, this effect represents a shift in behavior that can add up over time and across different situations, helping to build long-lasting independent learning habits.

The four-step process—getting support from others, forming beliefs, boosting motivation, and solidifying behavior—fits well with the ideas of both Social Cognitive Theory (SCT) and Self-Determination Theory (SDT).SCT highlights that the main role of support from the environment is to boost people’s confidence in their abilities, which is essential for them to get involved in activities [79]. In our findings, verbal encouragement, behavioral modeling, and emotional affirmation from parents, teachers, and peers significantly enhanced adolescents’ confidence in their exercise capabilities (β = 0.41, p < .001), demonstrating effective transfer from contextual input to personal belief [80].

SDT further posits that perceived competence is a necessary precursor to intrinsic motivation. When adolescents believe they are capable, they are more likely to experience physical activity as self-endorsed and fulfilling [81]. Our data confirmed this: self-efficacy strongly predicted intrinsic motivation (β = 0.27, p < .001). This qualitative shift from external obligation to internal endorsement lays the foundation for sustained engagement. Additionally, the final link in the chain—exercise motivation → autonomous physical learning—was also robust (β = 0.31, p < .001), aligning with meta-analytic evidence suggesting that intrinsic motivation has a stronger predictive effect on persistence and self-regulation than extrinsic forms of motivation [82].

Even though the chain mediation explains only a small part of the overall effect, it is important because it shows how support from others changes into personal behavior. Social support is not directly absorbed; rather, it is cognitively and motivationally processed by the learner before being converted into sustained, self-directed action. Based on this pathway, educational programs should follow three steps: first, the initiation phase, where social support quickly boosts self-confidence through encouragement and examples; second, the transformation phase, where supportive experiences improve motivation; and third, the consolidation phase, where gradually increasing challenges and teamwork help create lasting habits. These steps ensure that contextual resources are fully internalized as lifelong motivational assets, reinforcing adolescents’ autonomous engagement in physical activity.

### 5.5. Moderating effect of gender on the relationship between social support and physical learning

This study further examined whether the sequential mediation pathway from social support to autonomous physical learning behavior varied by gender. Results from the multi-group SEM revealed a significant moderating effect, thereby confirming Hypothesis 5. Specifically, the indirect link from social support to exercise motivation through self-efficacy (SS → SE → EM) was much stronger for female students (β = 0.127, 95% CI [0.104, 0.152], f² ≈ 0.02) than for male students (β = 0.088, 95% CI [0.070, 0.109], f² ≈ 0.01). While both paths reached statistical significance, they represent small practical effects. For instance, a one-standard-deviation increase in perceived social support led to a 0.127-standard-deviation increase in intrinsic motivation through self-efficacy among girls—consistent with a “small” effect as classified by Cohen. Nonetheless, such modest differences are meaningful when considering cumulative developmental effects over time, particularly in educational settings [83].

From a theoretical perspective, these findings are consistent with the propositions of both Social Cognitive Theory (SCT) and Self-Determination Theory (SDT). SCT emphasizes that self-efficacy, shaped by social modeling and feedback, plays a central role in initiating and sustaining behavioral engagement. Female adolescents, who often demonstrate greater sensitivity to interpersonal input, may be more likely to internalize supportive social cues into efficacy beliefs [84]. SDT further highlights that the satisfaction of competence and relatedness needs fosters intrinsic motivation. For girls, social support may more effectively satisfy these needs, enhancing motivational quality and persistence. The difference seen between genders in how motivation is influenced likely shows that girls are better at turning social support into motivation [85].

Even though the confidence intervals of other mediation pathways were similar, indicating no major gender differences outside the SS → SE → EM path, the overall effects were still a bit higher for female students.The total indirect effect was β = 0.347 for females and β = 0.310 for males, and the total effect was also greater among girls (β = 0.553) compared to boys (β = 0.481), resulting in a modest difference of Δβ = 0.072. These results imply that, in addition to enhancing self-beliefs, social support may strengthen female students’ resilience and sustained engagement by activating deeper motivational mechanisms. In contrast, male students may rely more directly on external task value, goal clarity, or autonomy-supportive cues, engaging less through internal belief-driven processes.

Practically, these findings drive home the importance of gender-responsive intervention strategies.For female students, programs should focus on strengthening self-efficacy through structured mastery experiences, individualized positive feedback, and peer modeling, thereby maximizing the motivational return from social support. For male students, more effective strategies may involve emphasizing clear goal structures, fostering autonomy, and providing performance-based reinforcement—approaches that align more closely with their motivational orientation. Designing gender-sensitive environments that address these differences will help maximize the effectiveness of physical education initiatives aimed at fostering autonomous physical learning among adolescents.

### 5.6. Pathway differences across latent types of autonomous physical learning

Latent Profile Analysis (LPA) identified four distinct behavioral subgroups among middle school students: Highly Engaged, Positively Regulated, Selectively Participative, and Passively Participative. These groups, which differ in how motivated and self-disciplined they are, support Hypothesis 6 by showing that there are important differences in how teenagers learn on their own in physical activities. To see if the ways social support affects behavior are different for each group, multi-group Structural Equation Modeling (SEM) was used. Measurement invariance was established (ΔCFI = 0.016; ΔRMSEA < 0.015), permitting valid cross-profile comparisons. The results showed that the way social support influences behavior varies between different groups, meaning that how effective social support is depends on the specific group.

In the Positively Regulated and Selectively Participative groups, the full sequential mediation pathway (SS → SE → EM → APL) was significant. Among Positively Regulated students, the chain path showed a standardized indirect effect of β = 0.074 (95% CI [0.051, 0.101], f² ≈ 0.02), while in Selectively Participative students, it was β = 0.066 (95% CI [0.042, 0.089], f² ≈ 0.02). These small but important effects show that social support helps build beliefs and motivation, which aligns with Self-Determination Theory (SDT), suggesting that meeting needs for competence and autonomy boosts intrinsic motivation [86]. These groups may represent adolescents who are moderately self-directed yet still reliant on contextual reinforcement to initiate and sustain active learning behavior [87].

In contrast, students in the Passively Participative group showed a weaker pattern. The chain mediation effect was β = 0.026 (95% CI [0.011, 0.042], f² ≈ 0.01), and the full pathway was only marginally significant. Even though their motivation and engagement were low, the maintained mediation pathway suggests that some internal ability can still be activated when there is support from outside. This matches earlier studies that show how outside support can help motivate less engaged teenagers, especially by offering emotional safety, clear expectations, and a structured environment. While these effects are modest, they underscore the equity-enhancing potential of social support in reaching disengaged students.

The Highly Engaged group presented a distinct pattern. Despite exhibiting the highest self-efficacy and motivation scores, this group showed no significant indirect or direct effects from social support. All path coefficients linking external support to outcomes were non-significant (β < 0.03, p > 0.10). This evidence that their behavior is sustained by fully internalized mechanisms, requiring minimal external prompting. Such students are likely guided by internal goals, identity integration, and self-regulatory habits, consistent with advanced stages of autonomous functioning. These findings indicate a ceiling effect: once adolescents reach a high level of motivational autonomy, external social inputs have limited incremental effect [88].

Taken together, these results highlight that autonomous physical learning operates through profile-specific psychological pathways. Social support is most effective in moderately motivated students, where it triggers belief formation and internal drive. For low-engagement students, its role is compensatory—establishing baseline participation [89]. For highly autonomous students, social support has little behavioral leverage. These findings improve our understanding of educational theories by highlighting that different students need different types of support and back recent suggestions in educational psychology for customized teaching methods based on student types [90].

From an applied perspective, these findings suggest the need for differentiated, profile-responsive educational strategies. For the Passively Participative group, early-stage interventions should focus on providing emotional scaffolding, structured peer interaction, and simplified mastery experiences to initiate motivation. For Positively Regulated and Selectively Participative students, programs should build on existing self-efficacy through autonomy-supportive feedback, explicit goal-setting, and progress tracking. For the Highly Engaged group, interventions should emphasize advanced challenges, personal growth opportunities, and autonomy-maximizing conditions to avoid plateauing. Tailoring strategies in this way will help maximize the impact of social support and foster lifelong engagement in physical activity.

## 6. Implications and limitations

The present investigation illuminates the key mechanisms through which social support fosters adolescents’ autonomous physical learning. By combining Social Cognitive Theory (SCT) and Self-Determination Theory (SDT), the study shows that social support encourages independent behavior in adolescents directly and also indirectly by boosting their confidence and motivation to exercise, following a specific sequence. The results also show that how strong these psychological pathways are depends on gender and individual behavior, highlighting the need to take personal differences into account when creating interventions.

From a practical standpoint, these findings offer concrete guidance for physical educators, school counselors, and policymakers. Interventions should prioritize strengthening perceived support from parents, teachers, and peers to enhance students’ belief in their capabilities and foster sustained intrinsic motivation. Educational programs should adopt differentiated strategies aligned with student profiles. For example, Passively Participative students may require emotional scaffolding and structured guidance to initiate engagement, while Positively Regulated and Selectively Participative students may benefit from goal-oriented feedback and autonomy-supportive learning environments. Highly Engaged students, by contrast, may require personalized development pathways that maintain motivational depth through advanced challenges. Schools are encouraged to implement behavioral screening tools to classify student types and allocate support accordingly. At the policy level, physical education curricula should adopt modular and tiered frameworks that allow educators to flexibly align instructional content with students’ motivational readiness and self-regulatory profiles.

Despite these contributions, several limitations warrant consideration. First, this study employed a cross-sectional design and relied solely on self-reported measures of social support, self-efficacy, and motivation, which may be subject to social desirability and recall biases. Future research should employ longitudinal or experimental designs to better infer causality and examine how changes in perceived support and internal belief systems influence behavior over time. Additionally, integrating objective indicators—such as wearable fitness tracking data, peer-reported motivational ratings, or classroom observation metrics—could enhance data reliability and reduce single-source bias.

Second, although the model accounted for demographic variables and examined gender moderation, it did not control for other influential psychological or environmental confounders. Factors like feelings of depression, anxiety, stress from school, or the overall school environment might affect motivation and how engaged students are, and not including them could weaken the model’s ability to explain the results [91]. Future research could include these variables as moderators or covariates to refine the structural pathways.

Third, while LPA revealed meaningful behavioral subtypes, the classification was based solely on motivational and behavioral indicators. Other latent traits—such as students’ physical literacy, goal orientation, or mindset—could enrich subgroup differentiation and inform more precise educational strategies. Moreover, while we tested gender and profile-based moderation, we could clarify when and for whom social support mechanisms are most effective with more complex moderated mediation frameworks.

Fourth, the sample was limited to middle school students in China, which may restrict the generalizability of the findings. Cultural norms surrounding authority, autonomy, and peer interaction likely influence how students perceive and internalize social support. Prior research has suggested that collectivist orientations may amplify the effects of interpersonal support on motivation, whereas individualistic cultures may place greater emphasis on self-initiation [92]. Future studies should therefore replicate and validate this model across diverse sociocultural settings to test its cross-cultural robustness and identify culturally responsive adaptations. Cultural values and educational traditions often deeply shape motivational constructs and behavioral expectations in teacher–student interactions. To support contextually grounded interventions, future research is encouraged to culturally adapt and empirically validate structured frameworks such as the Teacher Motivating Behaviors (TMB) classification system developed by Ahmadi et al. Created through international Delphi consensus, the TMB system provides a comprehensive taxonomy of SDT-aligned teacher behaviors. Applying this framework in non-Western educational settings may improve intervention fidelity, uncover culturally specific motivational mechanisms, and enhance the global applicability of SDT-based educational programs [93].

Lastly, future studies should explore the sequencing and timing of interventions, particularly how tiered strategies—e.g., low-threshold engagement scaffolds for passive students and autonomy-building modules for highly motivated learners—can be optimized across school terms. Mixed-method designs combining quantitative models with qualitative interviews would reveal more about students’ motivational narratives, barriers to engagement, and perceived impact of contextual support, further informing the development of responsive, evidence-based physical education programs.

In summary, this study helps flesh out how adolescents internalize social support into self-regulated learning behavior and highlights how this process differs across student types. Future research should further validate these findings across time and contexts, building a foundation for inclusive, evidence-based, and developmentally sensitive physical education programs.

## 7. Conclusion

This study looks at how young people’s ability to learn on their own is affected by social support. It finds that social support helps them learn independently, both directly and indirectly, by boosting their self-confidence and motivation to exercise. The impact of this support varies by gender, with girls being more likely to turn support into motivation. Additionally, the study shows that different groups of students respond in unique ways to social support when it comes to their learning behavior. Furthermore, the strength of this chain pathway is moderated by gender, with female students showing stronger belief-based translation of support into motivation. In addition, latent profile analysis reveals that the internal pathways from social support to learning behavior differ significantly across behavioral subtypes, highlighting the heterogeneity in psychological responsiveness to contextual support.

These findings not only help connect the theories of SCT and SDT in understanding how teenagers learn but also lay the groundwork for creating fair, age-appropriate, and tailored physical education programs that meet the varied needs of students in actual school settings.

## Supporting information

S1 File Appendix tables. (DOC)
