# Correction: How does social support influence autonomous physical learning in adolescents? Evidence from a chain mediation and latent profile analysis

**DOI:** 10.1371/journal.pone.0335961

**Published:** 2025-10-31

**Authors:** Jiamin Zhu, Ziyou Huang, Xiaotong Yuan, Zhiyong Zhang, Xiaoping Meng

The fifth author, Xiaotong Yuan, is incorrectly noted as the corresponding author. The correct corresponding author is Xiaoping Meng. Xiaoping Meng’s email address is: xpmeng1@gmail.com.

The third and fifth authors are also listed out of order. The correct order is: Jiamin Zhu^^1^^, Ziyou Huang^^2^^, Xiaotong Yuan^^1^^, Zhiyong Zhang^^3^^, Xiaoping Meng^^3^^.

**1** Graduate School, Shandong Sport University, Jinan, China, **2** School of Physical Education, Anqing Normal University, Anqing, China, **3** Institute of Physical Education and Social Sciences, Shandong Sports University, Jinan, China.

The correct citation is: Zhu J, Huang Z, Yuan X, Zhang Z, Meng X (2025) How does social support influence autonomous physical learning in adolescents? Evidence from a chain mediation and latent profile analysis. PLoS One 20(7): e0327020. https://doi.org/10.1371/journal.pone.0327020.
