# Expression of Concern: How does social support influence autonomous physical learning in adolescents? Evidence from a chain mediation and latent profile analysis

**DOI:** 10.1371/journal.pone.0354426

**Published:** 2026-07-23

**Authors:** The PLOS One Editors

After this article [1,2] was published, the following concerns were noted:

- The article cited as Reference 91 was retracted before [1,2] was published.
- The articles cited as References 5 and 78 were retracted after [1,2] was published.
- The articles cited as References 68, 73-75, 78, and 91 do not appear to support the corresponding cited statements.

In light of the cumulative issues, the *PLOS One* Editors issue this Expression of Concern. Readers are advised to interpret the article [1,2] with caution.

## References

[1] Zhu J, Huang Z, Meng X, Zhang Z, Yuan X. How does social support influence autonomous physical learning in adolescents? Evidence from a chain mediation and latent profile analysis. PLoS One. 2025;20(7):e0327020. doi: 10.1371/journal.pone.0327020 40591704 PMC12212492

[2] Zhu J, Huang Z, Yuan X, Zhang Z, Meng X. Correction: How does social support influence autonomous physical learning in adolescents? Evidence from a chain mediation and latent profile analysis. PLoS One. 2025;20(10):e0335961. doi: 10.1371/journal.pone.0335961 41171694 PMC12578180

