# Supplementary material for: How does social support influence autonomous physical learning in adolescents? Evidence from a chain mediation and latent profile analysis
Source: PLoS One. 2025 Jul 1;20(7):e0327020. doi: 10.1371/journal.pone.0327020 (PMC12212492; doi:10.1371/journal.pone.0327020)
Supplement: S1 File — (DOC) [file pone.0327020.s001.doc]

Appendix Table A1: Measurement Invariance Testing for the Social Support Scale across Gender

|  | χ2(df) | CFI | TLI | RMSEA[90% CI] | △CFI | △TLI | △RMSEA |
| --- | --- | --- | --- | --- | --- | --- | --- |
| Configural | 780.496 (115) | 0.978 | 0.974 | 0.073 [0.068-0.078] |  |  |  |
| Metric | 804.732 (131) | 0.977 | 0.973 | 0.074 [0.069-0.079] | 0.001 | 0.001 | 0.001 |
| Scalar | 847.910 (146) | 0.975 | 0.971 | 0.075 [0.071-0.080] | 0.002 | 0.002 | 0.001 |

Appendix Table A2: Measurement Invariance Testing for the Self‐Efficacy Scale across Gender

|  | χ2(df) | CFI | TLI | RMSEA[90% CI] | △CFI | △TLI | △RMSEA |
| --- | --- | --- | --- | --- | --- | --- | --- |
| Configural | 472.278(67) | 0.979 | 0.971 | 0.072[0.066,0.078] |  |  |  |
| Metric | 489.134(76) | 0.977 | 0.970 | 0.073[0.067,0.079] | 0.002 | 0.001 | 0.001 |
| Scalar | 654.210(80) | 0.973 | 0.966 | 0.075[0.069,0.081] | 0.004 | 0.004 | 0.002 |

Appendix Table A3: Measurement Invariance Testing for the Exercise Motivation Scale across Gender

|  | χ2(df) | CFI | TLI | RMSEA[90% CI] | △CFI | △TLI | △RMSEA |
| --- | --- | --- | --- | --- | --- | --- | --- |
| Configural | 1287.192(150) | 0.963 | 0.948 | 0.080 [0.076,0.084] |  |  |  |
| Metric | 1350.607(165) | 0.958 | 0.946 | 0.079[0.075, 0.083] | 0.005 | 0.002 | 0.001 |
| Scalar | 1465.312(180) | 0.953 | 0.942 | 0.080[0.077, 0.084] | 0.005 | 0.004 | 0.001 |

Appendix Table A4.

| Path | Male (n=1209) | Female(n=1150) | group differenceΔχ² | Conclusion |
| --- | --- | --- | --- | --- |
| SS → S-E | 0.49 (p < .001) | 0.53 (p < .001) | 2.31 (ns) | No significant |
| S-E → EM | 0.18 (p < .001) | 0.24 (p < .001) | **4.82 * ** | Female > Male |
| EM → APLB | 0.25 (p < .001) | 0.20 (p < .001) | 3.07 (ns) | No significant difference |
| SS → EM | 0.27 (**p** < .001) | 0.26 (**p** < .001) | 0.11 (ns) | No significant difference |
| SS → APLB | 0.31 (**p** < .001) | 0.35 (**p** < .001) | 2.96 (ns) | No significant difference |
| S-E → APLB | 0.14 (**p** < .001) | 0.17 (**p** < .001) | 2.44 (ns) | No significant difference |
